# Curriculum Innovation: Design and Implementation of Synchronous and Asynchronous Curricula to Enhance Residents' EEG Knowledge and Experience

**DOI:** 10.1212/NE9.0000000000200101

**Published:** 2023-11-14

**Authors:** Andres Fernandez, Jeremy J. Moeller, Dana B. Harrar, Rejean M. Guerriero, Jay Pathmanathan, Nitin Agarwal, Jennifer Madan Cohen, Amy Kephart, Fred A. Lado, Kinshuk Sahaya, Daniel J. Weber

**Affiliations:** From the Department of Neurology (A.F.), Sidney Kimmel Medical College at Thomas Jefferson University, Philadelphia, PA; Department of Neurology (J.J.M.), Yale School of Medicine, New Haven, CT; Department of Neurology (D.B.H.), Children's National Medical Center, Washington, DC; Department of Neurology (R.M.G.), Washington University School of Medicine and St. Louis Childrens Hospital, MO; Department of Neurology (J.P.), University of Pennsylvania, Philadelphia; Minnesota Epilepsy Group, P.A. (N.A., K.S.), Roseville, MN; Division of Pediatric Neurology (J.M.C.), Connecticut Childrens, University of Connecticut School of Medicine, Hartford, CT; American Epilepsy Society (A.K.), Chicago, IL; Department of Neurology (F.A.L.), Zucker School of Medicine at Hofstra/Northwell, Great Neck, NY; and Department of Neurology (D.J.W.), St. Louis University, MO.

## Abstract

**Introduction and Problem Statement:**

There is a need for structured EEG education opportunities to enhance neurology resident education. To address this need, the American Epilepsy Society (AES) supported the development and implementation of both synchronous and asynchronous EEG courses.

**Objectives:**

To produce EEG curricula that enhance resident EEG learning, increase interest in EEG and improve participants' knowledge, and to ensure that courses were highly used and available to the broadest range of learners.

**Methods and Curriculum Description:**

A multi-institutional group of EEG educators developed both courses. The synchronous curriculum consisted of a mixture of brief “mini-lectures” and interactive small group activities with self-assessment quizzes at the start and end of the course. The online asynchronous EEG curriculum consisted of self-directed slide sets, multiple-choice self-assessment quizzes and a structured EEG self-assessment tool. Courses were evaluated using postcourse surveys, analysis of pretest and posttest data, and analysis of user data from the asynchronous curriculum.

**Results and Assessment Data:**

Between 2019 and 2021, 56 residents participated in the synchronous EEG courses. On the resident survey, mean Likert scores for course design, planning, and learning outcomes ranged from 4.6 to 5.0 for the in-person courses and from 3.9 to 4.5 for the virtual course. On the 24-item pretests and posttests, overall median scores increased from 60% (14.5/24) to 75% (18/24; *p* < 0.001). More than 2,300 learners completed the first submodule of the asynchronous curriculum, but only 164 completed all sections. Most of those who completed the asynchronous curriculum reported that it was effective and appropriate for resident-level learning.

**Discussion and Lessons Learned:**

The AES EEG courses provide EEG learning opportunities for neurology residents beyond what is available at their home institutions. There is evidence for the effectiveness of the synchronous course, but the scope is limited to a small number of attendees. The asynchronous curriculum is more broadly available, but very few learners completed all elements. Future steps will include expansion of the in-person synchronous course and providing guidance to learners about the core and optional components of the asynchronous curriculum to increase the impact of both educational offerings.

## Introduction and Problem Statement

The ability to interpret an EEG is considered a core clinical skill for neurologists. Graduating residents are expected to interpret EEG studies without further training, even though a substantial percentage are likely not adequately trained.^1^ The current state of EEG education leaves graduating adult neurology residents with low confidence in their ability to independently interpret even routine EEGs.^1^

There is significant variability among neurology residency programs regarding methods of instruction, EEG exposure, and protected time for learning EEG.^2^ Furthermore, there are no mandates for minimum training experience, board certification, or fellowship training required for EEG interpretation. A recent survey indicated that residencies range from no dedicated EEG months to 4 months during a 4-year program.^2^ During these months, the amount of exposure to EEG can also vary, with a third of programs indicating 0–20 EEGs being read during a typical month.^2^

Typical approaches to EEG education include didactic lectures on basic concepts and supervised EEG review. Less commonly, dedicated EEG or neurophysiology courses are offered by programs. National organizations have created epilepsy curricula and retreats for residents, such as the Pellock Seminar at the Child Neurology Society or the Penry Mini-Fellowships. Still, these focus on clinical epilepsy and not on EEG. The International League Against Epilepsy (ILAE) academy, launched in July 2020, provides an interactive online structured approach to epilepsy and EEG education, including online EEG and MRI readers, but the focus is not exclusively on EEG.^3,4^

Recognizing these issues, in 2019, the American Epilepsy Society (AES) supported the development and implementation of an in-person (synchronous) EEG course hosted at the AES Annual Meeting for selected residents interested in epilepsy practice. Subsequently, it supported the development and launch of a freely available online asynchronous EEG curriculum that would provide educational resources in EEG for all residents and other interested learners.

## Objectives

Our goal was to produce curricula that would enhance EEG training at residents' home institutions, increase resident interest in EEG, and improve participants' knowledge of core concepts in EEG that should be understood at the end of residency. By developing both synchronous and asynchronous courses, we also wanted to ensure that the courses were engaging, highly used, and available to the broadest possible range of EEG learners.

## Methods and Curriculum Description

### Synchronous EEG Course

In 2017–2018, 2 of the authors, D.J.W. and K.S., discussed proposals with the AES to develop EEG-based educational content for neurology trainees. Further discussion identified a need for both synchronous and asynchronous educational resources. The AES established a workgroup. The initial workgroup members (D.J.W., K.S., A.F., J.J.M., and R.M.G.) were identified by the course codirectors as individuals with expertise in teaching EEG to residents and consisted of 5 practicing adult and pediatric epileptologists and electroencephalographers. The workgroup was later increased to 8 members (N.A., J.P., and Victoria Wong), through informal recruitment by the codirectors and committee members, for a final total of 2 pediatric and 6 adult EEG experts. Most of the committee members were in current academic practice or had prior experience in training residents in EEG. The workgroup started meeting in January 2019 with initial steps focused on creating a broad overview of the course structure.

#### Course Overview and Participants

To achieve the objective of enhancing residents' existing EEG education, the synchronous in-person (2019 and 2021) and synchronous virtual (2020) courses were planned for the first 1.5 days of the AES Annual Meeting, allowing trainees further exposure to epilepsy concepts and professionals during the remainder of the meeting. The AES called for nominations from child and adult residency program directors from across the United States through direct email, websites, and listservs. As an incentive for participation, AES offered residents a small travel stipend and complimentary registration to the annual meeting.

#### Course Structure and Educational Approach

The synchronous EEG course was designed to maximize engagement and simulate real-world EEG interpretation scenarios, with an approach rooted in adult learning theory.^5^ A pure lecture-based didactic method was thought to be particularly ill-suited to the teaching of a practical skill like EEG interpretation.^6^ Adult learners benefit most from material that is highly relevant to their workplace experiences. Therefore, there was a focus on clinical application of EEG in the settings that most residents would encounter this diagnostic test. Because of published data on the limited and highly variable EEG learning among residents,^1,2,7^ materials were designed with the assumption of very little preexisting knowledge among the learners. Readiness for the educational activity was achieved by delivering brief (10–15 minutes) lectures followed immediately by a small group activity (SGA) led by expert facilitators and centered around interactive quizzes or a group review of a short EEG file. Small groups consisted of 5 learners, and these groups remained together throughout the course to optimize the benefits of a shared learning experience.^8^ For many of the SGAs, we used EEG files (truncated to 10–15 minutes duration for the purposes of a short learning activity), opened in EEG software so that the group could manipulate data in real-world conditions (e.g., manipulating filters, sensitivity, and montages).

The workgroup members were divided into subgroups of 2–3, tasked with developing learning objectives for each section of the course. The subgroups worked on peer review of didactic educational material, interactive SGA educational content, and pretest and posttest items. After the course's first iteration, an additional subgroup planned networking opportunities for participants.

The content outline was based on consensus discussion among committee members using an iterative process. Consistent with our goal to enhance existing training, the outline was also informed by committee members' EEG curricula at their home institutions and by the Neurology Milestones.^9^ While the ILAE road map for competency-based education was not used as a source for our outline (this report was published after the committee meetings in January 2019 when we drafted our outline), there is substantial overlap between the EEG objectives in this road map and our topic outline.^3,4^

#### Course Delivery

The workgroup invited faculty members from across the country to contribute didactic content and to serve as lecturers for the course. Each selected faculty member submitted an outline of proposed content for the assigned lecture topic(s). The peer-review subgroup reviewed the proposed content to ensure consistency with the overall course structure.

After the faculty submitted the content (mini-lecture slides) and assessment material (multiple-choice questions [MCQs]), the material was again reviewed and approved for final use by the relevant subgroup. No faculty member was assigned more than 2 lecture topics to ensure diversity of experience and teaching styles.

The course structure is described in Table 1. Two (and rarely 3) mini-lectures alternated with interactive small group activities (20–30 minutes) or breaks. Networking was included in an unstructured format during the breaks and SGA sessions. An interactive group quiz at the end of the in-person sessions in 2019 and 2021 introduced a gamification aspect to the learning.

**Table 1:**
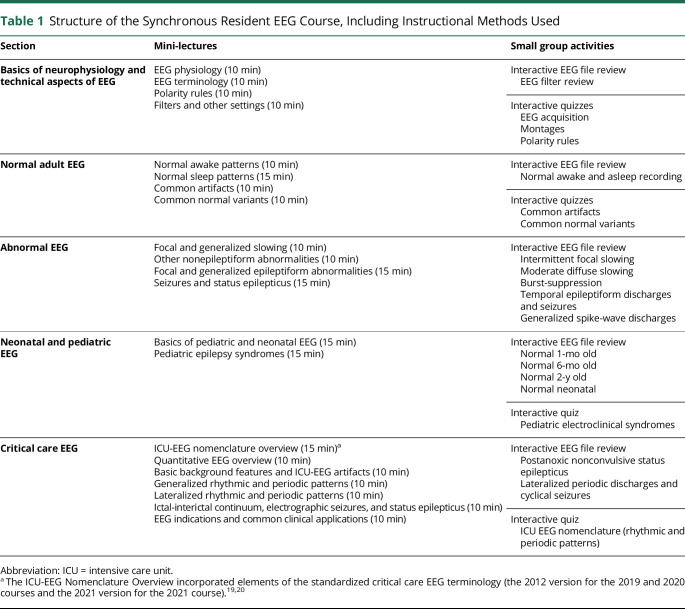
Structure of the Synchronous Resident EEG Course, Including Instructional Methods Used

Section	Mini-lectures	Small group activities
Basics of neurophysiology and technical aspects of EEG	EEG physiology (10 min) EEG terminology (10 min) Polarity rules (10 min) Filters and other settings (10 min)	Interactive EEG file review EEG filter review
		Interactive quizzes EEG acquisition Montages Polarity rules
Normal adult EEG	Normal awake patterns (10 min) Normal sleep patterns (15 min) Common artifacts (10 min) Common normal variants (10 min)	Interactive EEG file review Normal awake and asleep recording
		Interactive quizzes Common artifacts Common normal variants
Abnormal EEG	Focal and generalized slowing (10 min) Other nonepileptiform abnormalities (10 min) Focal and generalized epileptiform abnormalities (15 min) Seizures and status epilepticus (15 min)	Interactive EEG file review Intermittent focal slowing Moderate diffuse slowing Burst-suppression Temporal epileptiform discharges and seizures Generalized spike-wave discharges
Neonatal and pediatric EEG	Basics of pediatric and neonatal EEG (15 min) Pediatric epilepsy syndromes (15 min)	Interactive EEG file review Normal 1-mo old Normal 6-mo old Normal 2-y old Normal neonatal
		Interactive quiz Pediatric electroclinical syndromes
Critical care EEG	ICU-EEG nomenclature overview (15 min)^a^ Quantitative EEG overview (10 min) Basic background features and ICU-EEG artifacts (10 min) Generalized rhythmic and periodic patterns (10 min) Lateralized rhythmic and periodic patterns (10 min) Ictal-interictal continuum, electrographic seizures, and status epilepticus (10 min) EEG indications and common clinical applications (10 min)	Interactive EEG file review Postanoxic nonconvulsive status epilepticus Lateralized periodic discharges and cyclical seizures
		Interactive quiz ICU EEG nomenclature (rhythmic and periodic patterns)

Abbreviation: ICU = intensive care unit.

a The ICU-EEG Nomenclature Overview incorporated elements of the standardized critical care EEG terminology (the 2012 version for the 2019 and 2020 courses and the 2021 version for the 2021 course).^19,20^

Due to the coronavirus disease 2019 (COVID-19) pandemic, the AES Annual Meeting 2020 was held virtually. The synchronous virtual course was delivered in a single day. The same materials and instructional approaches were used. The same general structure was maintained, but prerecorded mini-lectures were provided to attendees ahead of the virtual course. The small group activities were conducted with breakout rooms. Faculty were asked to install EEG review software and data samples on their local computers (with a backup system of sequential EEG screenshots embedded into a PowerPoint file). Facilitators were encouraged to engage learners using best practices for online small group learning (e.g., cameras on, actively engaging participants, summarizing, and highlighting learning points).^10^

### Online Asynchronous EEG Curriculum

The development of the online asynchronous EEG learning curriculum (the American Epilepsy Society EEG Learning Curriculum [AESELC]) started in 2020, and the full curriculum was launched in March 2021. The AESELC was designed to be stand-alone, modular, structured, and self-paced. The AESELC development process was similar to the synchronous course. The structure and content outline was developed by consensus using a similar process to the synchronous course and again based on local EEG curricula and the EEG milestones. The content was created by 20 faculty members, including workgroup members, faculty from the synchronous course, and 5 additional invited faculty with experience in EEG education.

The AESELC is intended for a wider audience. It was divided into 9 major modules. Each module has several submodules consisting of a mini-lecture, 5 multiple-choice self-assessment items, and a structured EEG (self-) assessment tool (SEAT). The mini-lectures consist of slide sets for the learner to follow without a facilitator. The MCQ items were created by the faculty member responsible for the mini-lecture and then reviewed by 3 faculty members (A.F., J.J.M., and J.P.) for accuracy and quality. Each SEAT was linked directly to the preceding submodule (e.g., a module on neonatal EEG would be followed by a neonatal EEG SEAT) and consisted of images of a 1- to 2-minute segment of EEG recording in at least 2 different montages. Each faculty member then created an “answer key” based on a standard coding sheet, using a similar process to a previously published study by 2 of the committee members (J.P. and D.J.W.).^11^ The coding sheet included descriptions of state, background features, technical apsects, and both epileptic and nonepileptic abnormalities. Each coding sheet was subsequently reviewed by an independent committee of 3 of the authors (A.F., J.P., and D.J.W.), and the answer key was finalized by consensus. As learners progressed through each submodule, they were provided with immediate feedback on the MCQ items (including the breakdown of responses by other learners) and on how their SEAT responses matched with the answer key. An example of the SEAT format is shown in eAppendix 1 (links.lww.com/NE9/A53). In total, there are 9 courses with a total of 40 submodules with 200 MCQs, 40 lectures, and 40 SEATs.

For the online asynchronous curriculum, we used several principles of multimedia learning, including minimizing extraneous materials in the slides (Coherence principle), allowing learners to control the pace of the learning, chunking the activities into smaller segments (Segmenting principle), and including advance organizers and overviews of the curricular structure (Signaling principle).^12^ For the assessment, the multiple-choice items were developed by content experts according to the best practices of the National Board of Medical Examiners.^13^ They included immediate feedback and references for further reading to engage the retrieval effect in learning.^14^

### Program Evaluation

Our program evaluation process was designed to evaluate our ability to achieve the objectives of enhancing existing EEG training, increasing resident interest in EEG and improving participants' knowledge.

To evaluate engagement and interest, we sent postcourse surveys to each resident participant and faculty member. Respondents rated their agreement on a 5-point Likert scale (1 = strongly disagree, 5 = strongly agree) with items that focused on course design, learning, and career/professional variables. We asked faculty to rate their agreement with items focused on the mini-lectures, small group activities, and quizzes. The survey items varied slightly from year to year, so the analysis was performed only on items that remained unchanged between years.

We used survey data to modify subsequent synchronous courses and inform the transition to the virtual course format in 2020. We reviewed the qualitative comments from faculty and resident surveys identifying recurring themes in feedback.

For knowledge assessment in the synchronous course, we used a 24-item MCQ examination as a learner assessment method precourse and postcourse. Each of the faculty members was asked to write 3 multiple-choice items related to their mini-lecture content, so that the content outline of the assessment tool matched the overall composition of the pretest and posttest. Two workgroup members edited the items and constructed the final test on Qualtrics (2019 course), which was later migrated to Survey Monkey (2020 and 2021 courses). The same test was administered precourse and postcourse. The pretest was completed before the start of the EEG course, and the posttest was administered immediately after the didactic elements of the course ended.

Evaluation of engagement and utilization for the online asynchronous EEG curriculum included analysis of number of registered users and number of users who completed the entire curriculum. As a marker of usership of the individual submodules, we tracked the number of users who completed each posttest. As a quality measure, we also calculated the difficulty index for each posttest item (provided by the learning management system; Oasis LMS, Chicago, IL). To evaluate the achievement of primary program objectives, individuals who completed the entire course completed a postcourse survey focusing on the effectiveness of the format and the appropriateness of the format for level of learner.

### Statistical Analyses

All statistical calculations were performed using SPSS version 28.0.0.0 for Mac. Comparisons between pretest and posttest scores were performed using the Wilcoxon signed rank test. Comparisons of test scores between years were performed using single-factor analysis of variance. Comparisons of resident and faculty survey responses were performed using the Pearson χ^2^ test.

### Standard Protocol Approvals, Registrations, and Patient Consents

Our program evaluation methods were reviewed by a representative of the institutional review board (IRB) of Thomas Jefferson University and were determined not to represent IRB-regulated human research on the basis that the surveys and assessments focused on factual information about the learning process and did not gather additional personal information (i.e., information that is not otherwise publicly available) about living individuals. Confidentiality was maintained, and individual performance on pretests and posttests was not released.

### Data Availability

Anonymized data not published within this article or in the supplemental materials will be made available to qualified investigators on request.

## Results and Assessment Data

### Resident Selection and Participation

The first in-person synchronous course was held in 2019 during the AES Annual Meeting in Baltimore, MD. The second course was delivered virtually in 2020, and the third course returned to an in-person format at the 2021 AES Annual Meeting in Chicago, IL.

Twenty resident participants were selected from 124, 95, and 130 nominations in 2019, 2020, and 2021, respectively. Of the 20 chosen residents, 20, 17, and 19 attended in 2019, 2020, and 2021, respectively (with 1 person attending only day 2 in 2019).

For the virtual course in 2020, the total duration of prerecorded lectures was 250 minutes, but the median time participants watched the content was 122.2 minutes (ranging from 2.4 to 247.2 minutes). Some residents also participated via mobile devices, limiting the adequate review of EEG samples.

### Knowledge Assessment

**Figure 1:**
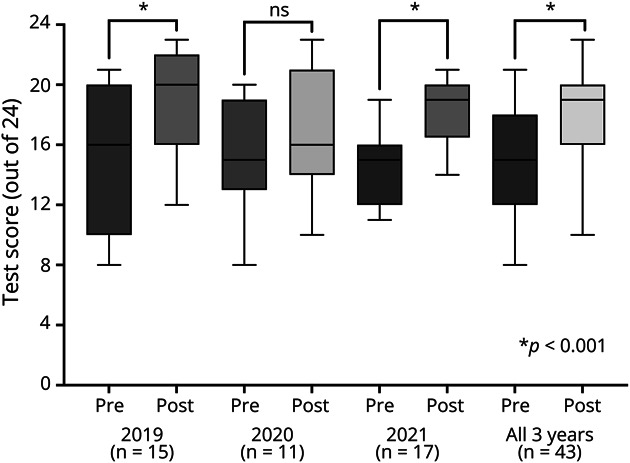
Box and Whisker Plot Indicating Participant Scores on the 24-Item Pretests and Posttests for the Synchronous Resident EEG Course, 2019–2021 The horizontal lines inside each box indicate the median scores for each iteration of the test, and the whiskers indicate the full range of scores. *p* Value was calculated using a Wilcoxon signed rank test. Only participants who had complete data for both the pretest and posttest were included in the analysis.

The results of pretests and posttests are shown in Figure 1. Among participants over all 3 years, there was an increase in the median score from 60% (14.5/24) to 75% (18/24; *p* < 0.001). There was a significant increase in scores after course completion for the in-person courses in 2019 and 2021, but no significant change for the 2020 virtual course. There was no significant difference in pretest or posttest scores between years of administration of the course.

### Survey Evaluation of Effectiveness of the Courses

The results of the resident postcourse survey are summarized in Table 2. The completion rate of the resident survey was 65% (13/20) in 2019, 76% (13/17) in 2020, and 84% (16/19) in 2021. Regarding our primary outcomes, most of the residents agreed that the course was a helpful addition to EEG training at the home institution and increased interest in EEG. Most of the respondents also agreed or strongly agreed that the course was well organized, had a good mixture of activities, and would be recommended to others. The in-person courses (2019 and 2021) had higher overall ratings than the virtual course (2020) for most elements of course design and learning outcomes.

**Table 2:**
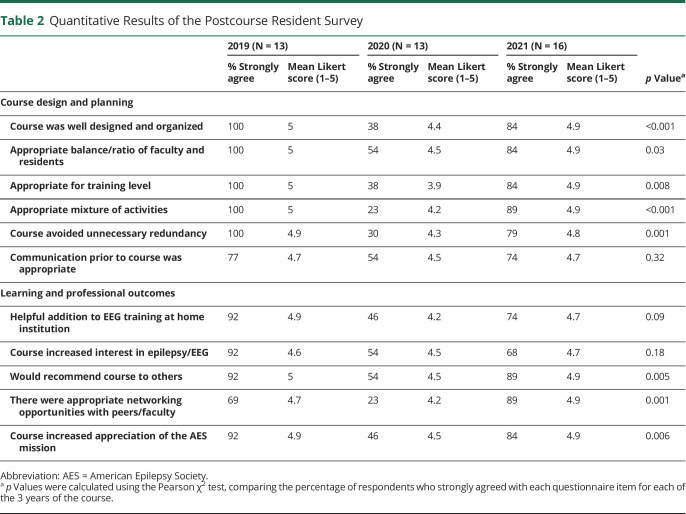
Quantitative Results of the Postcourse Resident Survey

	2019 (N = 13)		2020 (N = 13)		2021 (N = 16)		*p* Value^a^
	% Strongly agree	Mean Likert score (1–5)	% Strongly agree	Mean Likert score (1–5)	% Strongly agree	Mean Likert score (1–5)	
Course design and planning							
Course was well designed and organized	100	5	38	4.4	84	4.9	<0.001
Appropriate balance/ratio of faculty and residents	100	5	54	4.5	84	4.9	0.03
Appropriate for training level	100	5	38	3.9	84	4.9	0.008
Appropriate mixture of activities	100	5	23	4.2	89	4.9	<0.001
Course avoided unnecessary redundancy	100	4.9	30	4.3	79	4.8	0.001
Communication prior to course was appropriate	77	4.7	54	4.5	74	4.7	0.32
Learning and professional outcomes							
Helpful addition to EEG training at home institution	92	4.9	46	4.2	74	4.7	0.09
Course increased interest in epilepsy/EEG	92	4.6	54	4.5	68	4.7	0.18
Would recommend course to others	92	5	54	4.5	89	4.9	0.005
There were appropriate networking opportunities with peers/faculty	69	4.7	23	4.2	89	4.9	0.001
Course increased appreciation of the AES mission	92	4.9	46	4.5	84	4.9	0.006

Abbreviation: AES = American Epilepsy Society.

a *p* Values were calculated using the Pearson χ^2^ test, comparing the percentage of respondents who strongly agreed with each questionnaire item for each of the 3 years of the course.

The results of the faculty postcourse survey are summarized in Table 3. The completion rate was 80% (12/15) in 2019, 73% (11/15) in 2020, and 60% in (9/15) in 2021. The ratings for the in-person courses were overall higher than those for the virtual course. These differences reached statistical significance for the categories of the mixture of activities, level of engagement, and the number of small group activities.

**Table 3:**
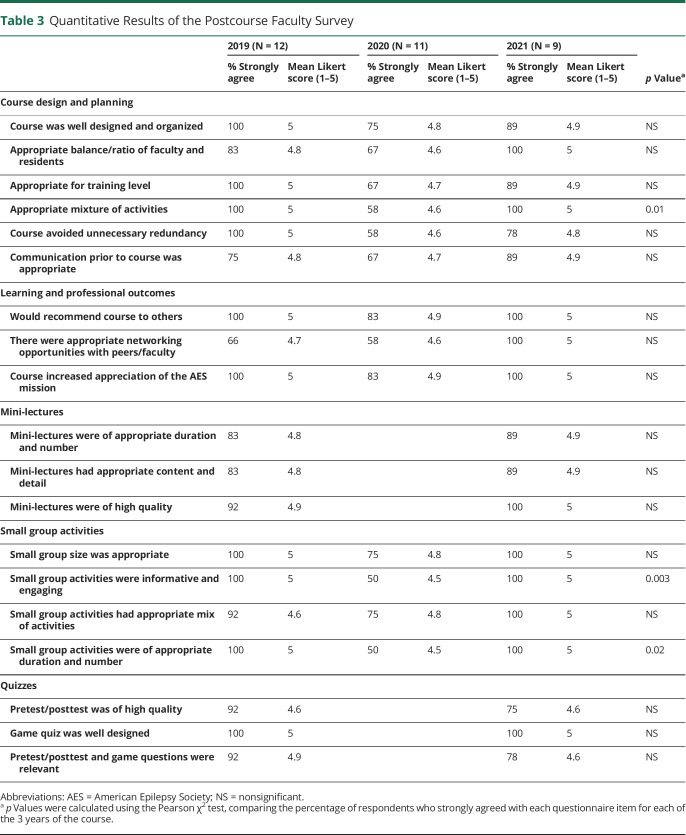
Quantitative Results of the Postcourse Faculty Survey

	2019 (N = 12)		2020 (N = 11)		2021 (N = 9)		*p* Value^a^
	% Strongly agree	Mean Likert score (1–5)	% Strongly agree	Mean Likert score (1–5)	% Strongly agree	Mean Likert score (1–5)	
Course design and planning							
Course was well designed and organized	100	5	75	4.8	89	4.9	NS
Appropriate balance/ratio of faculty and residents	83	4.8	67	4.6	100	5	NS
Appropriate for training level	100	5	67	4.7	89	4.9	NS
Appropriate mixture of activities	100	5	58	4.6	100	5	0.01
Course avoided unnecessary redundancy	100	5	58	4.6	78	4.8	NS
Communication prior to course was appropriate	75	4.8	67	4.7	89	4.9	NS
Learning and professional outcomes							
Would recommend course to others	100	5	83	4.9	100	5	NS
There were appropriate networking opportunities with peers/faculty	66	4.7	58	4.6	100	5	NS
Course increased appreciation of the AES mission	100	5	83	4.9	100	5	NS
Mini-lectures							
Mini-lectures were of appropriate duration and number	83	4.8			89	4.9	NS
Mini-lectures had appropriate content and detail	83	4.8			89	4.9	NS
Mini-lectures were of high quality	92	4.9			100	5	NS
Small group activities							
Small group size was appropriate	100	5	75	4.8	100	5	NS
Small group activities were informative and engaging	100	5	50	4.5	100	5	0.003
Small group activities had appropriate mix of activities	92	4.6	75	4.8	100	5	NS
Small group activities were of appropriate duration and number	100	5	50	4.5	100	5	0.02
Quizzes							
Pretest/posttest was of high quality	92	4.6			75	4.6	NS
Game quiz was well designed	100	5			100	5	NS
Pretest/posttest and game questions were relevant	92	4.9			78	4.6	NS

Abbreviations: AES = American Epilepsy Society; NS = nonsignificant.

a *p* Values were calculated using the Pearson χ^2^ test, comparing the percentage of respondents who strongly agreed with each questionnaire item for each of the 3 years of the course.

We found several recurring themes from the qualitative comments in the resident and faculty surveys (Table 4), including approval of the overall course design, especially the pacing and balance of short lectures with interactive small group activities. Participants also highlighted the benefit of networking opportunities. The participants noted that the virtual format did not work as well as the in-person format, including factors such as the assignment of videos for the mini-lectures not being as conducive to learning as the integration of mini-lectures during the in-person course.

**Table 4:**
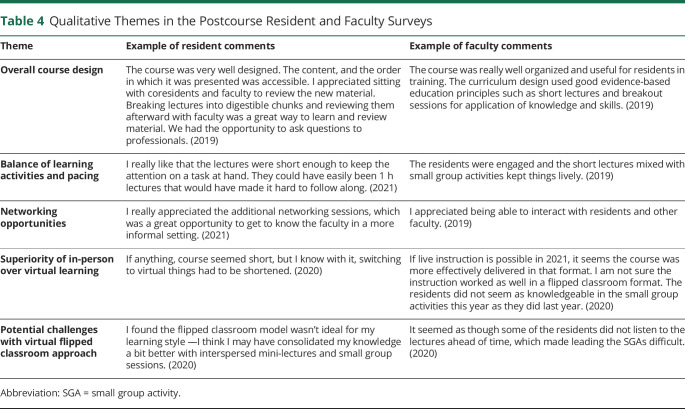
Qualitative Themes in the Postcourse Resident and Faculty Surveys

Theme	Example of resident comments	Example of faculty comments
Overall course design	The course was very well designed. The content, and the order in which it was presented was accessible. I appreciated sitting with coresidents and faculty to review the new material. Breaking lectures into digestible chunks and reviewing them afterward with faculty was a great way to learn and review material. We had the opportunity to ask questions to professionals. (2019)	The course was really well organized and useful for residents in training. The curriculum design used good evidence-based education principles such as short lectures and breakout sessions for application of knowledge and skills. (2019)
Balance of learning activities and pacing	I really like that the lectures were short enough to keep the attention on a task at hand. They could have easily been 1 h lectures that would have made it hard to follow along. (2021)	The residents were engaged and the short lectures mixed with small group activities kept things lively. (2019)
Networking opportunities	I really appreciated the additional networking sessions, which was a great opportunity to get to know the faculty in a more informal setting. (2021)	I appreciated being able to interact with residents and other faculty. (2019)
Superiority of in-person over virtual learning	If anything, course seemed short, but I know with it, switching to virtual things had to be shortened. (2020)	If live instruction is possible in 2021, it seems the course was more effectively delivered in that format. I am not sure the instruction worked as well in a flipped classroom format. The residents did not seem as knowledgeable in the small group activities this year as they did last year. (2020)
Potential challenges with virtual flipped classroom approach	I found the flipped classroom model wasn't ideal for my learning style —I think I may have consolidated my knowledge a bit better with interspersed mini-lectures and small group sessions. (2020)	It seemed as though some of the residents did not listen to the lectures ahead of time, which made leading the SGAs difficult. (2020)

Abbreviation: SGA = small group activity.

### Asynchronous Curriculum Evaluation

As of August 2023, 5,880 individuals had registered for the AESELC. The largest groups of registrants were residents/fellows (24%) and neurologists (21%). Other participants included students, other types of physicians, nurse practitioners, physician assistants, and EEG technologists. Thirteen percent of users did not indicate an occupation or level of training.

**Figure 2:**
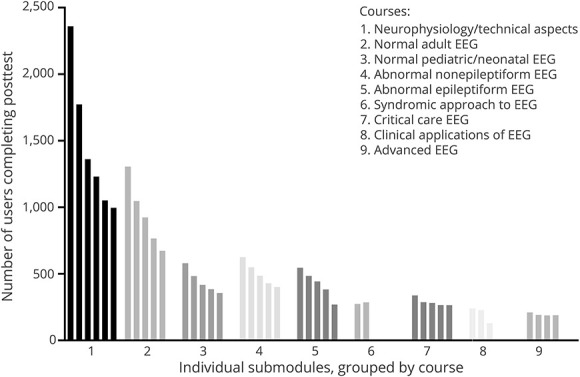
Number of Users Who Completed the Posttest for Each Submodule in the American Epilepsy Society EEG Learning Curriculum The submodules are grouped by course number, and the courses are organized in the order they appear in the curriculum.

As a measure of engagement and utilization for the asynchronous curriculum, Figure 2 shows the number of users who had completed the posttest for each of the individual submodules, grouped by course. More than 2,300 individuals completed the first submodule, but this number dropped to approximately 1,000 by the end of the first course. Each successive course had a lower number of individuals completing the first submodule, and through each of courses 2 through 5, retention of learners typically dropped by half. Learner retention was better in the later courses, but only 200–300 learners started each of these courses. The mean difficulty index (percent correct) for the posttest items was 0.74 (range 0.14–0.99), indicating good overall performance on the posttests.

A total of 55 individuals who completed the entirety of the course by April 2022 were asked to complete a postcourse survey. The respondents generally agreed the format was effective for the presentation of the EEG material (86% agree or strongly agree). The respondents agreed the course was most appropriate for the level of a resident (29, 53%) or fellow (16, 29%). As a proxy for improved knowledge, respondents overwhelmingly agreed that the course improved their ability to identify various components of the EEG from 78% (coma patterns) to 95% (focal slowing and spikes and sharp waves). Most (78%) agreed that the AESELC was as effective or more effective than other EEG learning modalities they had used and that they would recommend this course to others (91%). According to the unstructured comments, strengths included the accessibility of the course, the comprehensive nature of the course, and the novel SEAT. Weaknesses included a desire for the lectures to have an accompanying voice-over and a concern that the course may be difficult for individuals without any EEG experience. When asked for suggestions for improvement, 36 of 55 (66%) said nothing to improve, and the most common recommendation was to add voice-overs for the lectures (n = 5; 9%).

## Discussion and Lessons Learned

The synchronous resident EEG course and the online asynchronous EEG curriculum provide a structured, consensus-based approach to EEG education to complement EEG learning at residents' home institutions. We believe that there are several sources of evidence that we achieved our objectives to increase resident interest in EEG, improve resident EEG knowledge, and create highly engaging and broadly used resources that are available to many EEG learners.

### Synchronous Courses

The AES Resident EEG course was perceived by residents to be well designed and organized, appropriate to training level and a helpful addition to EEG training at residents' home institution. Individual components of the course, including the mini-lectures, small group activities, and pretests and posttests were perceived to be well balanced, of high quality, and relevant to learning. There was a modest but significant improvement in performance on an EEG knowledge assessment for the 2 in-person iterations of the course, but not for the virtual version. Overall, resident and faculty responses for the in-person versions of the EEG course were more positive than those for the virtual version. The face-to-face interaction during the in-person course also facilitated networking and further learning during informal discussions, and these opportunities were highly rated by both faculty and residents.

The virtual EEG course in 2020 still provided some immersion and engagement. A potential benefit of the virtual format is the ability to present to a larger audience because there are no travel costs and there was more flexibility for scheduling. However, despite the higher costs, the in-person format was preferred in relation to all major objectives, including learning, engagement, and networking. There may be several reasons for these findings. Residents may have had less protected time to participate and engage in these sessions, including potential increased local distractions and competing responsibilities. In addition, this course took place less than a year into the COVID-19 pandemic, at a time of substantial disruption in the clinical experiences of residents. It is possible that the competing personal and professional demands of the pandemic introduced distractions unrelated to the virtual format. Many residents did not watch the recorded lectures before the small group activities, and this may have contributed to the perceptions among residents and faculty of a lower level of preparation and engagement. While all residents were new to the course during the virtual year, the faculty had all experienced the synchronous course the year earlier, allowing them to form relationships with the faculty group and deliver the material in person at least once. This may have contributed to the fact that faculty members had an overall more positive impression of the virtual synchronous course than the resident participants.

### Online Asynchronous Curriculum

Although different from synchronous EEG courses, the online curriculum provides wider self-directed access to EEG content. Limitations include the lack of engagement and networking opportunities compared with the synchronous format. While many users completed the first 1–2 courses, only a small percentage completed the entire curriculum. This low long-term retention may suggest that the linear structure of the course is a weakness. There may be a missed opportunity to engage learners in “core” elements of EEG early in the course, leaving some technical aspects (e.g., history of EEG, physiology, and technology) to later in the course, for more committed learners. In addition, it could be possible to create “tracks” based on learner type (e.g., pediatric vs adult neurology residents, technologists, students, etc).

The development and implementation of the AESELC had unique challenges. The biggest challenge has been technologic. Unlike the synchronous course, there is no readily available mechanism for the online delivery of electronic EEG recordings for dynamic review and interpretation. The “mini-lectures” in the AESELC consist of static slides with some animation but without voice-over. This was chosen to ensure the self-navigable structure, but at the same time, it compromises some aspects of effective multimedia learning. More sophisticated learning management systems may offer better delivery mechanisms. The variability in styles of interpretation of EEG was also a challenge in the development of the SEAT. The reference interpretation used in the curriculum was achieved by consensus, but that may not always be possible for all elements of EEG.

### Assessment and Program Evaluation

There are inherent limitations in the pretest/posttest format of learner assessment.^15^ The pretests and posttests were identical, so some of the improvement could be attributed to the effects of repeated retrieval rather than learning, although the learners did not get any feedback after completing the pretest. In addition, the EEG images in the test were static and are much simpler than the range of findings that are encountered in actual practice. However, learner assessment was not the principal objective of the EEG courses. As outlined earlier, our goals also included providing tools to enhance EEG training that occurs in residents' home programs and to develop curricula that are engaging and used by a broad range of learners. The need for more detailed outcome assessment instruments was identified with the experience gathered with the design and implementation of the EEG courses. Challenges include the need for more substantial resources to collect and analyze the data of more complex instruments of learner assessment, including longitudinal follow-up. From the program evaluation perspective, information on how the learner uses the knowledge obtained in the course would provide valuable data to modify the course to meet learners' needs better and understand the different contexts of EEG teaching and learning across various institutions.

### EEG Courses in the Context of Residency EEG Education

Currently, each residency program must provide its own EEG teaching, leading to a high degree of variability in experiences. Digital tools can allow for sharing of learning resources between institutions (including those developed for the EEG course), reducing the amount of effort that local faculty have to spend on the most fundamental concepts so that local teaching time is engaged in helping residents hone their skills in an authentic clinical setting.^16^ Based on our experiences, the small group sessions were essential to the learning process and were very well received by both faculty and residents in postcourse surveys. This highlights an opportunity to integrate these instructional methods into resident EEG learning experiences at home institutions. These experiences were also the seed of the interactive components of the online asynchronous EEG curriculum, including a short quiz and a SEAT at the end of each module to replicate the immediate feedback that occurred during the synchronous course.

No course will be able to replace hands-on EEG reading.^17^ It will be up to the residency program and the local experts at each site to partner with the learner to apply the knowledge gained in the course to the real-world clinical setting. As such, developing a standardized national course does not replace the need for local experts nor does it eliminate practice variability.

### Future Directions

We have shown evidence of the feasibility, effectiveness, and acceptability of tools to augment EEG learning at home institutions, and our goal is to ensure that these tools are used by the broadest range of learners. For the synchronous course, the in-person iterations were more effective than the virtual version, but they are resource-intensive and will always have a limited scope. We plan to continue the in-person course, expanding attendance to 40 residents per year and focusing on individuals with an interest in epilepsy and EEG. In this way, we can leverage the effectiveness of this course in increasing interest in EEG/epilepsy careers and providing networking opportunities for high-interest learners.

The online asynchronous EEG curriculum has broader reach, but our analysis shows less engagement, with few individuals completing every module. Early modules are highly used, and there is an opportunity to provide a road map for resident use of this course, with designated core and optional components and “tracks” for different learners. We could also educate residency directors on how to integrate these modules into their formal curricula, possibly providing residents with protected time to complete them. Similar issues with course completion have been seen in other online asynchronous curricula for neurology residents, although because of a self-paced curriculum, learners will complete components at all times of day and at vastly different rates; some completing all components in a few hours while others spread the experience over several months.^18^ The asynchronous curriculum is simply a tool and could be better used with the engagement of stakeholders in resident EEG education at individual institutions. Ultimately, there is the opportunity to curate this and other resources into a set of consensus-based didactics and proposed rotation structures for resident EEG education.

## Appendix Authors

**Table:**

Name	Location	Contribution
Andres Fernandez, MD, MSEd	Department of Neurology, Sidney Kimmel Medical College at Thomas Jefferson University	Drafting/revision of the article for content, including medical writing for content; major role in the acquisition of data; study concept or design; and analysis or interpretation of data
Jeremy J. Moeller, MD, FRCPC	Department of Neurology, Yale School of Medicine	Drafting/revision of the article for content, including medical writing for content; major role in the acquisition of data; study concept or design; and analysis or interpretation of data
Dana B. Harrar, MD, PhD	Department of Neurology, Children's National Medical Center	Drafting/revision of the article for content, including medical writing for content; study concept or design
Rejean M. Guerriero, DO	Department of Neurology, Washington University School of Medicine and St. Louis Children's Hospital	Drafting/revision of the article for content, including medical writing for content; study concept or design
Jay Pathmanathan, MD, PhD	Department of Neurology, University of Pennsylvania	Drafting/revision of the article for content, including medical writing for content; major role in the acquisition of data; and study concept or design
Nitin Agarwal, MD, FAES	Minnesota Epilepsy Group, P.A.	Drafting/revision of the article for content, including medical writing for content; study concept or design
Jennifer Madan Cohen, MD, FAES	Division of Pediatric Neurology, Connecticut Children's, University of Connecticut School of Medicine	Drafting/revision of the article for content, including medical writing for content; major role in the acquisition of data
Amy Kephart, MPH, CAE	American Epilepsy Society	Major role in the acquisition of data; study concept or design
Fred A. Lado, MD, PhD	Department of Neurology, Zucker School of Medicine at Hofstra/Northwell	Study concept or design
Kinshuk Sahaya, MD, MBA	Minnesota Epilepsy Group, P.A.	Drafting/revision of the article for content, including medical writing for content; study concept or design
Daniel J. Weber, DO	Department of Neurology, St. Louis University	Drafting/revision of the article for content, including medical writing for content; major role in the acquisition of data; and study concept or design

## Glossary

AES: American Epilepsy Society
AESELC: AES EEG Learning Curriculum
COVID-19: coronavirus disease 2019
ILAE: International League Against Epilepsy
IRB: institutional review board
MCQ: multiple-choice question
SEAT: structured EEG self-assessment tool
SGA: small group activity
